# Screening for Autochthonous Phytoextractors in a Heavy Metal Contaminated Coal Mining Area

**DOI:** 10.3390/ijerph14091068

**Published:** 2017-09-15

**Authors:** Kuangjia Li, Zijian Lun, Lin Zhao, Qilong Zhu, Yansheng Gu, Manzhou Li

**Affiliations:** 1 School of Environmental Science and Engineering, Tianjin University, Tianjin 300072, China; kuangjiali@tju.edu.cn (K.L.); zhaolin@tju.edu.cn (L.Z.); 2 Henan Anhuan Environmental Sci-Tech Co., Ltd., Anyang 455000, China; lunzijian@2014.cug.edu.cn; 3 School of Environmental Studies, China University of Geosciences, Wuhan 430074, China; 4 CNOOC Research Institute, Beijing 100000, China; zhuql9@cnooc.com.cn; 5 State Key Laboratory of Biogeology and Environmental Geology, China University of Geosciences, Wuhan 430074, China; 6 Department of Land and Resources of Henan Province, Zhengzhou 450000, China; limz2006@126.com

**Keywords:** adaptability factor, gangue dump, heavy metal contamination, native phytoextractor, phytoremediation

## Abstract

In order to protect public health and crops from soil heavy metal (HM) contamination at a coal mining area in Henan, central China, HM pollution investigation and screening of autochthonous HM phytoextractors were conducted. The concentrations of cadmium (Cd), lead (Pb), copper (Cu) and zinc (Zn) in surface soils exceeded the corresponding local background values and the China National Standard (CNS). The maximum potential ecological risk (RI) was 627.30, indicating very high ecological risk. The monomial risk of Cd contributed the most to the RI, varying from 85.48% to 96.48%. The plant community structure in the study area was simple, and was composed of 24 families, 37 genera and 40 species. *B. pilosa*, *A. roxburghiana*, *A. argyi*, *A. hispidus* were found to be the most dominant species at considerable risk sites. Based on the comprehensive analysis of Cd concentration, bioconcentration factor, translocation factor and adaptability factor, *B. pilosa* and *A. argyi* had potential for phytoextraction at considerable risk sites. *A. roxburghiana* had potential for Cd phytoextraction at moderately risk sites and *A. hispidus* seemed suitable for phytostabilization. The results could contribute to the phytoremediation of the similar sites.

## 1. Introduction

The coal industry is one of the most important pillars of China’s economy. The coal mining activity produces a large amount of coal gangues, from which toxic HMs could be released during the destruction of gangue mineral structure under the combined effects of water, microorganisms, vegetation, sunlight radiation and heat [1]. The HMs, unlike organic pollutants, cannot be biodegraded or biodeteriorated. As a consequence, they accumulate in the environment. In addition, HMs from the environment also migrate toward and accumulate in the living organisms by the processes of bioaccumulation and biomagnification through the trophic levels of the ecosystem. The accumulation of HMs in agricultural soils and water bodies poses a considerable threat to human health by different exposure pathways—direct ingestion, dermal absorption, inhalation and food chains [2,3]. To deal with these problems, a variety of physical and chemical treatment approaches have been developed, such as in situ vitrification, soil incineration, excavation and landfill, soil washing, soil flushing, solidification, and stabilization of electrokinetic systems [4,5]. However, these techniques have certain drawbacks like high expense, intensive labor, secondary pollution and irreversible change of the soil properties [6]. As one of the novel remediation methods, phytoremediation is considered as a cost-effective and environmentally-friendly solution to the HM pollution problem.

Phytoremediation refers to the use of vegetation and associated soil microbes to lower the concentrations or hazardous effects of contaminants in the environments [7]. Generally, phytoremediation includes five techniques: phytoextraction (phytoaccumulation), phytofiltration, phytostabilization, phytovolatilization, and phytodegradation [8]. Phytoextraction is the HM absorbtion by plant roots, translocation and accumulation in above-ground (AG) biomass [9,10], which is the most useful phytoremediation technique for the removal of HMs from contaminated sediments or water [11,12]. In the process of phytoextractor selection, the native plants should be taken as a priority. The reasons are from the following points: (a) they tend to have higher viability [13]; (b) the autochthonous plants growing at contaminated sites are also considered as a valuable source of seed banks and gene pools [14]; (c) most importantly, alien plant species could reduce local plant species diversity [15,16,17] and pose non-negligible impacts at the local species, community and ecosystem level [18]. Many studies of HM phytoremediation with the native flora have been conducted [19,20].

The bioconcentration factor (BCF) and translocation factor (TF) are the most utilized indices for phytoremediation plant evaluation and selection [21]. However, they are not able to thoroughly characterize the phytoremediation ability of in situ plant species, because the phytoextraction efficiency depends on not only soil HM bioavailability and soil properties, but also the characteristics of the plant species like growth rate, AG biomass production, tolerance to the target HM toxicity, and adaptability to local environmental conditions [22,23,24]. Therefore, adaptability factor was introduced as an amendment of the screening criterion in this study. A typical coal gangue dump and the adjacent land in Jiaozuo, central China, were chosen as the study area. A previous study conducted at this same site showed that the gangue dump produced HM pollution in the adjacent cultivated land controlled by prevailing wind and posed a considerable threat to the environment [25]. In order to ensure the environmental safety during the reuse of the dump site and the crop safety of the surrounding farmland require systemic remediation and restoration. This study aimed to: (a) investigate and assess HM pollution and screen of target HM for phytoremediation. The metals evaluated are Cd, Pb, Cu, Cr and Zn; (b) survey local vegetation and determine the HM contents in different native species; (c) analyze the corresponding adaptability factors, bioconcentration factors and translocation factors in order to screen out suitable native plant species for phytoremediation of the target HM.

## 2. Materials and Methods

### 2.1. Site Description

**Figure 1:**
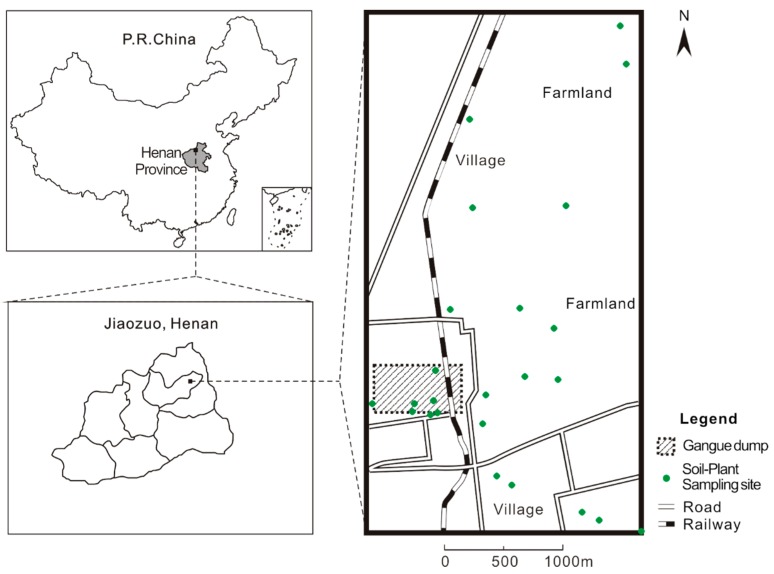
Map showing the research area and sampling sites.

The study area (N 35°16′2.1′′, E 113°21′8.2′′) is located in Yanmazhuang (Jiaozuo, central China, Figure 1). The elevation of the terrain gradually decreases from the northwest to the southeast; the direction of the groundwater flow is to the southeast (130°). The area is a part of the front Taihang piedmont alluvial-pluvial fan, which is under the control of a temperate continental monsoon climate. There are three types of soil: sandy loam, clay loam and silty clay loam. The groundwater depth varies between 1 m and 3 m. The prevailing wind directions are northeast and southwest. The annual average temperature, precipitation and average evaporation are 14 °C, 610 mm and 2039 mm, respectively. The main crops are summer maize (*Zea mays*) and winter wheat (*Triticum aestivum*).

### 2.2. Sample Collection and Analysis

The investigation was mainly carried out at the coal gangue dump site and the adjacent land, which covered around 30 km^2^. All sampling sites were chosen with caution to avoid anthropogenic disturbances such as the main roads, railways, etc. Thirteen surface soil/gangue and twenty-one plant samples were collected. The soil samples were collected at the depth of 3–10 cm with wooden chips. The sampling density varied from 50 to 500 m according their distance from the gangue dump. A higher sampling density was chosen closer to the dump. Each sample was collected in triplicate.

Each duplicate plant sample was formed with 3–5 different plant individuals and each duplicate soil sample was the mixture of the soil subsamples collected beneath the sampled plant individuals. Ten additional plant samples were also collected. Since these species were unlikely phytoextractors according to the literature review, duplicate sampling was not applied. The plant samples were separated into the above-ground part (shoots) and root parts. Representative samples for each species were selected for the herbaria and subsequent identification. GPS coordinates of all sampling sites were documented. Soil pH was measured by an IQ150 portable pH-conductivity meter (Spectrum Technologies, Inc., Aurora, IL, USA).

The plant samples were washed three times using distilled water and oven-dried. The samples were ground, sieved through 100 mesh sieve, and digested with an H_2_O_2_-HClO_4_-HF-HNO_3_ method in PTFE jars on an electric heating plate. Two Chinese national standard samples (GBW07427, GBW10048), as well as a blank sample was used as references to control the quality of the sample analysis. The total concentrations of cadmium (Cd), lead (Pb), copper (Cu), chromium (Cr), and zinc (Zn) in plants, soil and gangue were measured by inductively coupled plasma-atomic emission spectroscopy (ICP-AES, IRIS Intrepid II XSP, Thermo Scientific, Waltham, MA, USA). The recovery rates of the standard samples were within 90–110%.

### 2.3. Potential Ecological Risk Assessment

The concept of potential ecological risk assessment (RI) was introduced by Hakanson in 1980, and is widely utilized in soil and sediment HM studies. The calculation formulas are as follows:

(1) $$C_{f}^{i}\;=\;C_{D}^{i}/C_{R}^{i}$$

(2) $$E_{r}^{i}\;=\;T_{r}^{i}\;\times \;C_{f}^{i}$$

(3) $$RI\;=\;\overset{m}{\underset{i=1}{\sum }}E_{r}^{i}$$

*RI* is the sum of all indexes of five HMs, $C_{f}^{i}$ is the pollution factor, $C_{D}^{i}$ is the measured concentration, $C_{R}^{i}$ is a reference value, which is set as Henan HM background content (Table 1), $E_{r}^{i}$ is the monomial potential ecological risk index, and $T_{r}^{i}$ is the toxic factor for HM which is 30, 5, 5, 2 and 1 for Cd, Pb, Cu, Cr and Zn. Five categories of monomial risk are low potential ecological risk ($E_{r}^{i}$ < 40), moderate potential ecological risk (40 ≤ $E_{r}^{i}$ < 80), considerable potential ecological risk (80 ≤ $E_{r}^{i}$ < 160), high potential ecological risk (160 ≤ $E_{r}^{i}$ < 320) and very high ecological risk ($E_{r}^{i}$ ≥ 320). Four categories of RI value are low ecological risk (*RI* < 150), moderate ecological risk (150 ≤ *RI* < 300), considerable ecological risk (300 ≤ *RI* < 600) and very high ecological risk (*RI* ≥ 600) [26].

### 2.4. Screening of Phytoextractors

The prior objective of the remediation is to reduce the content of toxic HMs in soil. The following factors related to phytoremediation should be considered.

#### 2.4.1. BCF Calculation

BCF indicates the HM uptake efficiency of a plant species into its tissues from the surrounding environment [27]. It is calculated as follows [28]:

BCF = C_tissue_/C_soil_ (4)

where C_tissue_ is the concentration of the target HM in the plant harvested tissue and C_soil_ is the concentration of the same HM in the soil or other substrate.

#### 2.4.2. TF Calculation

TF indicates the efficiency of the plant in translocating the accumulated HM from its roots to shoots. It is calculated as follows [29]:

TF = C_AG_/C_root_ (5)

where C_AG_ is concentration of the HM in plant aboveground part and C_root_ is concentration of the HM in plant roots.

#### 2.4.3. Adaptability Factor

Adaptability factor indicates the dominance of the phytoextractor candidates at sites of different pollution levels. Two categories for pollution level are set-moderate risk site (MRS) for the soil RI under 300, considerable risk site (CRS) for the soil RI over 300. Three categories for dominance are set-rare (R, dominance < 50%), common (C, 50% < dominance < 75%), and dominant (D, dominance > 75%). Overall, four categories are set to evaluate the adaptability factor of the plants: (G1) the dominance of the plants was D at CRS sites; (G2) the dominance was C at CRS sites; (G3) stood for D dominance status at MRS sites; (G4) contained the rest of the plants, including the species of dominance status C at MRS sites and dominance status R.

### 2.5. Statistical Analyses

The mean values and standard deviation were calculated by using Microsoft Excel (Microsoft, Redmond, WA, USA). Statistical significance of differences among means was determined by one-way ANOVA using R language, taking *p* ≤ 0.05 as significant level and Tukey Contrasts multiple comparisons of means were carried out for pairwise analyses.

## 3. Results and Discussion

### 3.1. Soil HM Contamination

The concentration of the soil Cd, Pb and Zn exceeded the corresponding local soil HM background value (Table 2). The Cd concentration varied from 0.17 to 1.38 mg·kg^−1^. Five samples exceeded the level 1 China National Standard (CNS, GB15618-1995, Table 1) and six samples exceeded level 2 CNS. Cd concentration of all samples were higher than Henan soil background. The Pb concentration was 19.25–91.13 mg·kg^−1^. There are ten samples exceeded level 1 CNS and twelve exceeded Henan background [30]. The highest Zn concentration was 172.38 ± 30.93 mg·kg^−1^. Five sample exceeded level 1 CNS. The Cu and Cr concentration of all the samples were within level 1 CNS, while Cu concentration of several samples slightly exceeded the background value.

**Figure 2:**
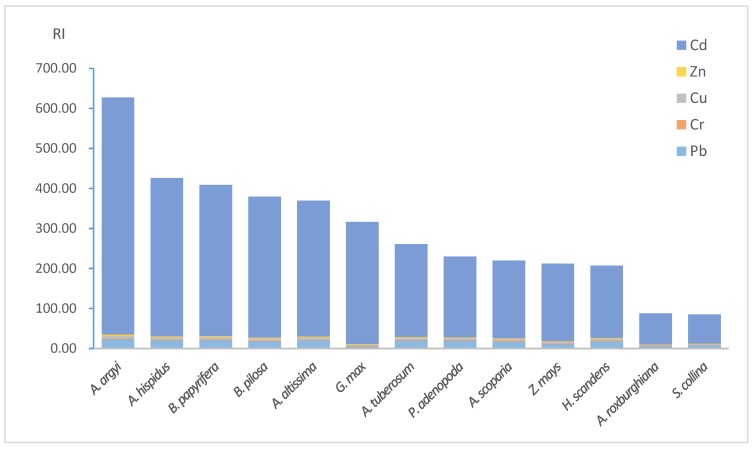
RI of the habitats of plant samples.

As shown in Figure 2, the highest RI was up to 627.30. There were 1, 5, 5 and 2 samples for very high risk, considerable risk, moderate risk and low risk, respectively. Figure 2 also shows that the contribution of Cd monomial risks varied from 85.48 percent to 96.48 percent with an average of 90.51 percent among all the samples. The results of potential ecological risk assessment indicated the gangue dump and the adjacent land was contaminated by Cd, Cu, Pb and Zn, and Cd contributed the most of the RI. Cd exposure could result in carcinogenicity, mutagenicity, and teratogenicity, endocrine disruption, biological calcium regulation interference, renal failure and chronic anemia [31,32,33]. In addition, the reference dose (RfD) of oral soil digestion is provided by USEPA and Leung et al. [34,35], which is 0.001, 0.003, 0.04, 0.0035 and 0.3 mg·(d·kg^−1^) for Cd, Cr, Cu, Pb and Zn. The RfD of Cd is much lower than those of the others. Also, some researchers stated that Cd is the HM of most concern because it is the “only metal that might pose human or animal health risks at plant tissue concentrations that are not generally phytotoxic” [36]. Along with the potential ecological index assessment result, Cd should therefore be considered as the target element for the screening of native phytoextractors.

### 3.2. Vegetation Species and HM Content

All plant herbaria were identified based on *Flora Reipublicae Popularis Sinicae* [37]. There were 24 familiae, 37 genera and 40 species, including wild plants and cultivated plants. Among all 40 species, there were seven meso-microphanerophytes, seven nanophanerophytes, 24 herbs and two vines. Compositae and Poaceae were the dominant familiae. Twenty-four species were perennial plants, 16 species were annual and biennial plants. The wild plants were mainly distributed on the east and south hilltop and hillside. The vertical structure of plant communities was simple, mostly consisted of 1 or 2 layer(s). The vertical structure at the foot of the gangue dump was single due to the effect of tractor shovel activities. Only herbs like *Salsola collina* Pall., *Bidens pilosa* Linn. *Artemisia argyi* Lévl. *et* Van. and *Artemisia scoparia* Waldst. Et Kit. could be found. The cultivated plants were mainly located at the farmland surrounding the gangue dump. Besides the plant species shown in Table 2, there were 17 additional plants in the study area. They were *Ulmus pumila* Linn., *Chenopodium album* Linn., *Amaranthus viridis* Linn., *Brassica pekinensis* (Lour.) Rupr., *Raphanus sativus* Linn., *Amygdalus persica* Linn., *Gossypium arboreum* Linn., *Cornus officinalis* Sieb. *et* Zucc., *Diospyros kaki* Thunb., *Periploca sepium* Bunge, *Pharbitis nil* (Linn.) Choisy, *Galium bungei* Steud., *Xanthium sibiricum* Patrin *ex* Widder, *Phragmites australis* (Cav.) Trin. *ex* Steud., *Chloris virgata* Sw., and *Sorghum bicolor* (Linn.) Moench These 17 species were not further studied because their dominance were relatively low, their habitats were away from the gangue dump, and there were few reports about them being potential phytoextractors.

The result of soil-root-AG HM determination was shown in Table 2, Table 3 and Table 4. The Cd content in AG of *Artemisia argyi* Lévl. *et* Van., *Artemisia scoparia* Waldst. *Et* Kit., *Bidens pilosa* Linn., and the root of *Artemisia argyi* Lévl. *et* Van. was higher than the normal range for plants (0.1–1 mg·kg^−1^) [38,39]. The Pb concentration of *Artemisia argyi* Lévl. *et* Van. AG was the highest among the studied species, which was 15.45 ± 2.62 mg·kg^−1^. The Zn concentration of *Artemisia scoparia* Waldst. sample was 446.25 ± 55.84 mg·kg^−1^, which was higher than the upper limit (400 mg·kg^−1^) for contaminated plants [40], near phytotoxic levels (500 mg·kg^−1^) [38,41].

### 3.3. Screening for Native Phytoextractors

BCF is a more important measure than shoot metal concentration when considering the potential of a given candidate species for phytoextraction [23]. As shown in Table 5, the plant samples with BCF greater than 1 were mainly for Cd element. The highest one was in AG sample of *Artemisia roxburghiana* Bess. (8.22), and the lowest one was in maize grain (0.27). Only a few samples had BCF higher than 1 for the other HM elements.

Accumulation and exclusion are two tolerance strategies evolved by plants to live in HM polluted medium [42,43,44,45]. HM accumulators are defined by the TF > 1, while root HM transport to shoot in excluders is restricted and TF < 1 [43,46,47]. A TF value greater than 1 indicates the translocation of HM from root to AG parts [48]. The translocation factors of the studied plant species were shown in Table 6. The Cd TFs of *Artemisia roxburghiana* Bess., *Artemisia scoparia* Waldst. *Et* Kit., *Bidens pilosa* Linn., and *Artemisia argyi* Lévl. *et* Van. were higher than 1. They could be considered as Cd accumulators. The other ones behaved as Cd excluders, suggesting Cd immobilization in the roots.

The dominance of species is a combination of abundance and cover of plants, which indicates the biomass and adaptability in certain habitats. With the introduction of the habitat classification by the potential ecological risk assessment, the adaptability factor can reflect the plants’ vitality in the environment under different levels of HM stress. The adaptability factor could be a useful supplementary criterion other than BCF and TF for phytoextractor screening from a community ecological point of view, though it is a “black box” of the plants’ adaptability indication, and lacks quantitative tolerance study like the response of biomass, length, chlorophyll (CHL), superoxide dismutase (SOD), peroxidase (POD), malondialdehyde (MDA), and soluble protein (SP) within the plants under HM stress. Furthermore, DNA changes determined by random amplified polymorphic DNA (RAPD) could to be employed as a useful tool to identify the genotoxic effects caused by HMs within plant individuals [49]. Therefore, the results obtained from the field survey should be validated with the methods mentioned above.

The previous study stated that adverse effects were observed in plant tissues stressed by high HM content, such as reduction of biomass, length and total protein contents [50]. In this study, all plant species in Table 2 did not show obvious symptom of phytotoxicity. They were divided into 4 categories (G1–G4) based on their dominance status and the pollution level of their habitats. (G1): the dominances of the plants were higher than 75% at CRS sites, which indicated high adaptability under intensive HM pollution stress. There were 5 species in this group: *Bidens pilosa* Linn., *Artemisia argyi* Lévl. *et* Van., *Artemisia scoparia* Waldst. *Et* Kit., *Humulus scandens* (Lour.) Merr., and *Arthraxon hispidus* (Trin.) Makino. (G2): the dominances were C (40–75%) at CRS sites. *Broussonetia papyrifera* (Linn.) L'Hér. *ex* Vent. belonged to this group. (G3) stood for D dominances status (>75%) at MRS sites, including *Salsola collina* Pall., *Artemisia roxburghiana* Bess., *Cynodon dactylon* (Linn.) Pers., *Setaria viridis* (Linn.) Beauv., and *Eleusine indica* (Linn.) Gaertn. These species had high adaptability under moderate HM stress. (G4) contained the rest of the plants, including the species of dominance status C at MRS sites and dominance status R.

Herbs are more promising for phytoextraction than shrubs and trees due to their high growth rate, high biomass, and more adaptability under stress [51]. Therefore, the following herbs like *Bidens pilosa* Linn., *Artemisia argyi* Lévl. *et* Van., *Artemisia scoparia* Waldst. *Et* Kit., *Artemisia roxburghiana* Bess., and *Arthraxon hispidus* (Trin.) Makino were chosen to be discussed based on bioconcentration factors, translocation factors and adaptability factors analyzed above.

*Bidens pilosa* Linn. belonged to G1. The Cd content, BCF and TF was 1.16 ± 0.30 mg·kg^−1^, 1.41, and 3.05, when the root soil concentration was 0.82 ± 0.57 mg·kg^−1^. According to Yoon et al. [10], plant species with both BCF and TF greater than 1 have the potential to be used for phytoextraction. *Bidens pilosa* met this criterion in this study. *Bidens pilosa* was considered as a hyperaccumulator under laboratory conditions in previous studies [52]. The Cd concentration of AG was higher than 100 mg·kg^−1^, the BF and TF values were all greater than 1.0, when soil Cd concentration was 8 and 16 mg·kg^−1^ in the form of CdCl_2_ solution. The roots were able to uptake HM from aqueous phase. However, soil HMs in the field were mainly insoluble and unavailable for uptake by plants due to strong binding of HM ions to soil particles and precipitation [4]. This could explain the BCF difference between present study and the laboratory experiment. In addition, the biomass showed no significant reduction and the HM stress did not show any significant impact on CHL, SOD, POD, MDA, and SP, which showed high tolerance to Cd [52]. The high adaptability at polluted sites, BCF, TF greater than 1, and the potential to be a hyperaccumulator suggested *Bidens pilosa* Linn. could be used as a native phytoextractor for soil Cd phytoremediation.

In the previous study [53], the AG content, BCF and TF of Cd in *Artemisia argyi* Lévl. *et* Van. were 35.5 ± 3.2 mg·kg^−1^, 3.55 and 3.11 in the soil treated by 10 mg·kg^−1^ for Cd (CdCl_2_·2.5H_2_O), 1000 mg·kg^−1^ for Pb (Pb(CH_3_COO)_2_·3H_2_O), 400 mg·kg^−1^ for Cu (CuSO_4_·5H_2_O) and 1000 mg·kg^−1^ for Zn (ZnSO_4_·7H_2_O). The biomass reduced significantly by about 50%. This suggested that *Artemisia argyi* Lévl. *et* Van. did not show hyperaccumulator characteristics and lacked strong tolerance under the high level of HM co-stress. In this study, *Artemisia argyi* Lévl. *et* Van. was classified as a G1 plant. Its Cd content, BCF and TF were 2.67 ± 0.69 mg·kg^−1^, 1.93 and 1.78. The results showed it could be considered as a potential phytoextractor. The difference of accumulation performance could be explained by the lower root soil Cd content as well as the lower bioavailability caused by the difference between the natural soil and the soil-HM compound formulated in the laboratory.

*Artemisia scoparia* Waldst. *Et* Kit. was reported having certain ability to extract HM from contaminated soil in previous research [54]. Its AG content, BCF and TF of Cd were 3.33 ± 0.55 mg·kg^−1^, 0.69 and 1.25 in the soil with Cd concentration of 4.76 ± 1.2 mg·kg^−1^ at a mine-affect area, which did not meet the requirement for phytoextractor due to the low BCF. According to another investigation [55], *Artemisia scoparia* Waldst. *Et* Kit. from different sites showed quite contradictory phytoextraction performances. The AG content, BCF and TF of the sample from site 3 were 4.02 ± 0.89 mg·kg^−1^, 10.05 and 4.14, while the parameter from site 4 were 0.34 ± 0.12 mg·kg^−1^, 1.31 and 0.79. The total soil Cd concentration were similar at two sites, which were 0.30 ± 0.04 for site 3 and 0.26 ± 0.05 mg·kg^−1^ for site 4. The difference between DTPA extractable Cd (0.04 ± 0.004 for site 3 and 0.01 ± 0.001 mg·kg^−1^ for site 4) suggested that the phytoextraction characteristics of *Artemisia scoparia* Waldst. *Et* Kit. relied on the DTPA extractable Cd in the soil and might lack the ability to mobilize carbonate-bound and residual state Cd. *Artemisia scoparia* Waldst. *Et* Kit. was divided into G1 group in present study. Its AG content, BCF and TF were 2.62 ± 0.16 mg·kg^−1^, 5.82 and 5.70, which had the potential as a phytoextractor on the sites with high bioavailable Cd content.

*Artemisia roxburghiana* Bess. belonged to G3 in this case. The soil pollution level was moderate: 23.96 ± 4.81, 12.25 ± 3.29, 52.55 ± 15.37 and 0.18 ± 0.06 mg·kg^−1^ for Pb, Cu, Zn and Cd. The BCF and TF of AG for Pb, Cu, Zn and Cd was 0.27 and 4.11, 1.57 and 2.85, 1.67 and 4.48, 8.22 and 6.73, respectively. The lack of dominance at CRS sites might limit its remediation application. Lei and Duan [56] found that *Artemisia roxburghiana* Bess.’s BCF for Pb, Zn and Cu were less than 0.005, and the HM content remained a low level at very highly polluted sites (over 7000, 35,000 and 400 mg·kg^−1^ for Pb, Zn and Cu), showing weak phytoremediation capacity. However, *Artemisia roxburghiana* Bess. was also found to have the ability to reduce a considerable amount of Pb, Zn and Cu concentration and increase the soil fertility in rhizosphere simultaneously. Although *Artemisia roxburghiana* Bess. was unfit for phytoextraction at considerable risk sites, it showed the potential to treat the soil at moderate risk sites due to the high BCF and TF as well as the ability of soil fertility improvement.

*Arthraxon hispidus* (Trin.) Makino was classified as one of G1 species in this study. The Cd content, BCF and TF of AG was 0.54 ± 0.05 mg·kg^−1^, 0.59 and 0.39. Our results were in consistency with the previous experiment conducted in Cd(NO_3_)_2_ treated soil with 3.00 mg·kg^−1^ of total Cd. The BCF and TF of AG was 4.17 and 0.95, also showing the exclusion characteristics [57]. The BCF difference between the two study could be explained by high bioavailable Cd content due to the implementation of Cd(NO_3_)_2_ during the sample formulation. The exclusion strategic of *Arthraxon hispidus* (Trin.) Makino suggested it had little potential for phytoextraction, but it had the potential to be used for phytostabilization at considerable risk sites.

## 4. Conclusions

The concentration of Cd, Pb, Cu and Zn at the study site exceeded the local background value and corresponding CNS, The RI values were over 300 in 46.2% of the sediment samples, the maximum RI was 627.80, which indicated very high ecological risk. Cd contributed the most to the RI, and was the main contaminant as well as the prior target for soil remediation. There were 24 familiae, 37 genera and 40 species of plants in the study area, and the community structure is simple. *Bidens pilosa* Linn. and *Artemisia* argyi Lévl. *et* Van. had high potential to be phytoextractors due to their high AG Cd concentration, BCF, TF and dominance at considerable risk sites. *Artemisia roxburghiana* Bess. was fit for Cd phytoextraction at moderately risk sites. *Arthraxon hispidus* (Trin.) Makino was classified as a Cd excluder, which seemed suitable for phytostabilization. Further studies should be conducted under controlled environment, such as the responses of plants’ BCF, TF, biomass, CHL, SOD, POD, MDA, SP and DNA changes, as well as their influence on the soil organic matter, pH, and HM bioavailability.

## Figures and Tables

**Table 1:** CNS and Henan background value of HMs (mg·kg^−1^).

	Pb	Cr	Cu	Zn	Cd
Backgroundin Henan	19.60 ± 4.62	63.80 ± 13.25	19.70 ± 4.80	60.10 ± 15.30	0.07 ± 0.02
CNS (GB15618-1995)	35 ^I^, 300 ^II^	90 ^I^	35 ^I^, 100 ^II^	100 ^I^	0.20 ^I^, 0.60 ^II^

^I^: level 1 CNS, ^II^: level 2 CNS (6.5 < pH < 7.5).

**Table 2:** Sediment heavy metal concentrations of the plants’ habitats (mg·kg^−1^).

Plants at the Sampling Location	Pb	Cr	Cu	Zn	Cd
*Zea mays* Linn. (H)	43.21 ± 18.55 ac	38.76 ± 11.69 a	20.61 ± 6.71 ab	62.18 ± 26.83 ab	0.35 ± 0.16 ab
*Arthraxon hispidus* (Trin.) Makino (H)	79.03 ± 12.10 cd	51.13 ± 9.28 a	26.83 ± 3.06 ab	130.23 ± 42.15 ab	0.92 ± 0.47 ab
*Bidens pilosa* Linn. (H)	69.1 ± 22.04b cd	32.75 ± 9.11 a	24.86 ± 5.02 ab	134.00 ± 38.38 ab	0.82 ± 0.57 ab
*Artemisia argyi* Lévl. *et* Van. (H)	91.13 ± 25.51 d	41.85 ± 8.07 a	29.88 ± 4.63 b	172.38 ± 30.93 b	1.38 ± 0.28 b
*Artemisia roxburghiana* Bess. (H)	23.96 ± 4.81 ab	28.28 ± 2.90 a	12.25 ± 3.29 a	52.55 ± 15.37 a	0.18 ± 0.06 a
*Artemisia scoparia* Waldst. *Et* Kit. (H)	66.93 ± 15.22 bcd	60.4 ± 22.58 a	23.77 ± 4.29 ab	88.08 ± 28.37 ab	0.45 ± 0.13 ab
*Salsola collina* Pall. (H)	28.36 ± 4.40 ab	30.13 ± 1.85 a	13.16 ± 0.91 ab	51.73 ± 0.82 a	0.17 ± 0.02 a
*Ailanthus altissima* (Mill.) Swingle (T)	78.59 ± 8.36 cd	51.46 ± 6.41 a	25.75 ± 2.76 ab	119.79 ± 35.06 ab	0.79 ± 0.39 ab
*Humulus scandens* (Lour.) Merr. (H)	69.67 ± 2.74 bcd	54.27 ± 6.14 a	23.15 ± 0.63 ab	89.94 ± 1.86 ab	0.42 ± 0.04 ab
*Glycine max* (Linn.) Merr. (H)	19.25 ± 4.72 a	48.52 ± 19.78 a	12.14 ± 11.62 a	97.19 ± 59.06	0.51 ± 0.41 ab
*Populus adenopoda* Maxim. (T)	75.05 ± 2.65 cd	50.13 ± 2.00 a	23.06 ± 0.54 ab	95.35 ± 3.55 ab	0.47 ± 0.09 ab
*Broussonetia papyrifera* (Linn.) L’Hér. *ex* Vent. (T)	81.77 ± 9.36 cd	44.99 ± 3.14 a	26.20 ± 3.68 ab	132.09 ± 40.29 ab	0.88 ± 0.50 ab
*Allium tuberosum* Rottler *ex* Sprengle (H)	77.05 ± 0.65 cd	52.02 ± 0.11 a	24.34 ± 0.75 ab	99.25 ± 0.35 ab	0.54 ± 0.01 ab
SD	26.59	14.05	7.32	45.82	0.45
Mean	61.78	44.97	22.00	101.90	0.60
CV	0.43	0.31	0.33	0.45	0.74

Values represent the mean ± standard error (*n* = 3). Values followed by the same letter within the column are not significantly different at *p* < 0.05 level. (H)—herb, (S)—shrub, (T)—tree, (V)—vine.

**Table 3:** Heavy metal concentrations of plant AG part (mg·kg^−1^).

Plants	Status	Habitat	Pb	Cr	Cu	Zn	Cd
*Zea mays* Linn. (H)	D	MRS	7.71 ± 2.16 b	23.27 ± 0.02 ac	20.32 ± 4.22 a	31.75 ± 9.38 a	0.50 ± 0.16 ab
*Arthraxon hispidus* (Trin.) Makino (H)	D	CRS	8.10 ± 0.19 b	34.93 ± 0.68 c	12.73 ± 2.28 a	56.00 ± 8.80 a	0.54 ± 0.05 ab
*Bidens pilosa* Linn. (H)	D	CRS	6.86 ± 0.56 b	23.42 ± 2.56 ac	12.95 ± 0.81 a	81.09 ± 21.71 a	1.16 ± 0.3 ab
*Artemisia argyi* Lévl. *et* Van. (H)	D	CRS	15.45 ± 2.62 c	29.05 ± 3.91 bc	19.13 ± 4.28 a	56.35 ± 9.37 a	2.67 ± 0.69 c
*Artemisia roxburghiana* Bess. (H)	D	MRS	6.45 ± 1.04 b	20.73 ± 2.49 ab	19.29 ± 3.72 a	87.68 ± 26.74 a	1.48 ± 0.33 b
*Artemisia scoparia* Waldst. *Et* Kit. (H)	D	CRS	14.01 ± 1.73 c	30.00 ± 7.51 bc	37.08 ± 13.52 b	446.25 ± 55.84 b	2.62 ± 0.16 c
*Salsola collina* Pall. (H)	D	MRS	0.59 ± 0.38 a	13.13 ± 0.23 a	5.92 ± 0.09 a	30.75 ± 3.65 a	0.34 ± 0.03 a
*Ailanthus altissima* (Mill.) Swingle (T)	R	CRS	10.58 ± 2.61 bc	29.70 ± 5.58 bc	10.40 ± 0.68 a	53.74 ± 24.04 a	0.76 ± 0.27 ab
*Humulus scandens* (Lour.) Merr. (H)	D	CRS	6.40 ± 1.25 ab	25.27 ± 5.73 ac	12.03 ± 0.16 a	58.59 ± 26.91 a	0.45 ± 0.16 a
*Glycine max* (Linn.) Merr. (H)	C	CRS	3.65 ± 0.52 a	32.70 ± 4.70 c	15.02 ± 2.29 a	47.8 ± 11.36 a	0.33 ± 0.14 a
*Populus adenopoda* Maxim. (T)	C	MRS	6.37 ± 0.40 b	29.35 ± 5.34 bc	12.80 ± 0.40 a	98.39 ± 60.59 a	0.74 ± 0.12 ab
*Broussonetia papyrifera* (Linn.) L’Hér. *ex* Vent. (T)	C	CRS	10.47 ± 2.52 bc	29.92 ± 1.94 bc	9.63 ± 2.03 a	36.46 ± 13.29 a	0.87 ± 0.34 ab
*Allium tuberosum* Rottler *ex* Sprengle (H)	C	MRS	5.51 ± 0.04 ab	35.36 ± 2.08 c	17.29 ± 1.19 a	88.3 ± 5.93 a	0.43 ± 0.09 a

Values represent the mean ± standard error (*n* = 3). Values followed by the same letter within the column are not significantly different at *p* < 0.05 level. (H)—herb, (S)—shrub, (T)—tree, (V)—vine.

**Table 4:** Heavy metal concentrations of plant root part (mg·kg^−1^).

Plants	Status	Habitat	Pb	Cr	Cu	Zn	Cd
*Zea mays* Linn. (H)	D	MRS	8.57 ± 3.77 bc	24.72 ± 2.85 a	13.01 ± 4.95 ab	63.13 ± 46.55 ab	0.66 ± 0.32 ab
*Arthraxon hispidus* (Trin.) Makino (H)	D	CRS	13.46 ± 2.7 c	27.98 ± 6.43 a	16.33 ± 3.73 b	51.73 ± 14.48 ab	1.40 ± 0.38 bc
*Bidens pilosa* Linn. (H)	D	CRS	2.96 ± 0.74 ab	25.61 ± 5.37 a	14.93 ± 2.18 ab	123.63 ± 29.73 b	0.38 ± 0.09 a
*Artemisia argyi* Lévl. *et* Van. (H)	D	CRS	5.71 ± 1.92 ab	25.5 ± 5.83 a	12.55 ± 2.43 ab	52.7 ± 11.85 ab	1.5 ± 0.33 c
*Artemisia roxburghiana* Bess. (H)	D	MRS	1.57 ± 0.37 ab	23.16 ± 4.83 a	6.76 ± 1.38 a	19.56 ± 3.44 a	0.22 ± 0.05 a
*Artemisia scoparia* Waldst. *Et* Kit. (H)	D	CRS	8.03 ± 2.42 bc	20.57 ± 2.36 a	9.35 ± 1.59 ab	47.85 ± 12.73 ab	0.46 ± 0.06 a
*Salsola collina* Pall. (H)	D	MRS	0.56 ± 0.14 a	14.67 ± 2.05a	8.39 ± 0.79 ab	45.78 ± 18.63 ab	0.85 ± 0.12 ac

Values represent the mean ± standard error (*n* = 3). Values followed by the same letter within the column are not significantly different at *p* < 0.05 level. (H)—herb, (S)—shrub, (T)—tree, (V)—vine.

**Table 5:** BCFs of Plants.

Plants	Sample Types	BCF Pb	BCF Cr	BCF Cu	BCF Zn	BCF Cd
*Zea mays* Linn.	leaf	0.18	0.60	0.99	0.51	1.11
	root	0.20	0.64	0.63	1.02	1.47
*Glycine max* (Linn.) Merr.	AG	0.19	0.67	1.24	0.49	0.46
*Arthraxon hispidus* (Trin.) Makino	AG	0.10	0.68	0.47	0.43	0.59
	root	0.17	0.55	0.61	0.40	1.52
*Ailanthus altissima* (Mill.) Swingle	leaf	0.13	0.58	0.40	0.45	0.96
*Bidens pilosa* Linn.	AG	0.10	0.72	0.52	0.61	1.41
	root	0.04	0.78	0.60	0.92	0.46
*Artemisia argyi* Lévl. *et* Van.	AG	0.17	0.69	0.64	0.33	1.93
	root	0.06	0.61	0.42	0.31	1.09
*Artemisia roxburghiana* Bess.	AG	0.27	0.73	1.57	1.67	8.22
	root	0.07	0.82	0.55	0.37	1.22
*Artemisia scoparia* Waldst. *Et* Kit.	AG	0.21	0.50	1.56	5.07	5.82
	root	0.12	0.34	0.39	0.54	1.02
*Populus adenopoda* Maxim.	leaf	0.08	0.59	0.56	1.03	1.57
*Broussonetia papyrifera* (Linn.) L’Hér. *ex* Vent.	leaf	0.13	0.67	0.37	0.28	0.99
*Salsola collina* Pall.	AG	0.02	0.44	0.45	0.59	2.00
	root	0.02	0.49	0.64	0.88	5.00
*Humulus scandens* (Lour.) Merr.	AG	0.09	0.47	0.52	0.65	1.07
*Allium tuberosum* Rottler *ex* Sprengle	AG	0.07	0.68	0.71	0.89	0.80

**Table 6:** TFs of Plants.

Plants	Sample Types	TF Pb	TF Cr	TF Cu	TF Zn	TF Cd
*Zea mays* Linn.	leaf	0.90	0.94	1.56	0.50	0.76
*Arthraxon hispidus* (Trin.) Makino	AG	0.60	1.25	0.78	1.08	0.39
*Bidens pilosa* Linn.	AG	2.32	0.91	0.87	0.66	3.05
*Artemisia argyi* Lévl. *et* Van.	AG	2.71	1.14	1.52	1.07	1.78
*Artemisia roxburghiana* Bess.	AG	4.11	0.90	2.85	4.48	6.73
*Artemisia scoparia* Waldst. *Et* Kit.	AG	1.74	1.46	3.97	9.33	5.70
*Salsola collina* Pall.	AG	1.05	0.90	0.71	0.67	0.40
